# Impacts of corn stover management and fertilizer application on soil nutrient availability and enzymatic activity

**DOI:** 10.1038/s41598-022-06042-9

**Published:** 2022-02-07

**Authors:** Fernando S. Galindo, Jeffrey S. Strock, Paulo H. Pagliari

**Affiliations:** 1 University of Sao Paulo, Centre for Nuclear Energy in Agriculture, Avenida Centenario, 303. CP 96, Piracicaba, CEP 13416-000 Brazil; 2 Department of Soil, Water, and Climate, University of Minnesota. Southwest Research and Outreach Center, 23669 130th, St. Lamberton, MN 56152 USA

**Keywords:** Plant sciences, Environmental sciences

## Abstract

Corn stover is a global resource used in many industrial sectors including bioenergy, fuel, and livestock operations. However, stover removal can negatively impact soil nutrient availability, especially nitrogen (N) and phosphorus (P), biological activity, and soil health. We evaluated the effects of corn stover management combined with N and P fertilization on soil quality, using soil chemical (nitrate, ammonium and Bray-1 P) and biological parameters (β-glucosidase, alkaline phosphatase, arylsulfatase activities and fluorescein diacetate hydrolysis—FDA). The experiment was performed on a Mollisol (Typic Endoaquoll) in a continuous corn system from 2013 to 2015 in Minnesota, USA. The treatments tested included six N rates (0 to 200 kg N ha^−1^), five P rates (0 to 100 kg P_2_O_5_ ha^−1^), and two residue management strategies (residue removed or incorporated) totalling 60 treatments. Corn stover management significantly impacted soil mineral-N forms and enzyme activity. In general, plots where residue was incorporated were found to have high NH_4_^+^ and enzyme activity compared to plots where residue was removed. In contrast, fields where residue was removed showed higher NO_3_^−^ than plots where residue was incorporated. Residue management had little effect on soil available P. Soil enzyme activity was affected by both nutrient and residue management. In most cases, activity of the enzymes measured in plots where residue was removed frequently showed a positive response to added N and P. In contrast, soil enzyme responses to applied N and P in plots where residue was incorporated were less evident. Soil available nutrients tended to decrease in plots where residue was removed compared with plots where residue was incorporated. In conclusion, stover removal was found to have significant potential to change soil chemical and biological properties and caution should be taken when significant amounts of stover are removed from continuous corn fields. The residue removal could decrease different enzymes related to C-cycle (β-glucosidase) and soil microbial activity (FDA) over continuous cropping seasons, impairing soil health.

## Introduction

The area used for corn (*Zea mays* L.) production comprises around 13% of the world's arable land and is expected to increase to over 190 Mha with yields surpassing 1.2 billion Mg per year by 2027^1,2^. According to the United States Department of Agriculture, the United States (US) produced above than 360 Mt of corn annually in the last five years, accounting for 32% of global corn production^3^ with more than 80% of its production occurring in the US Midwest. Corn has many uses, including human food and livestock feed as well as biofuel. In addition, ethanol production in the US is predominately derived from corn grain^4^.

The use of corn stover as a renewable energy could reduce greenhouse gas emissions (GHG) from transportation (fuel) with biofuel and reduced production of fossil fuels. However, the removal of corn stover could increase the carbon footprints/GHG emissions in the process of manufacturing fertilizer and increased application in the field to fulfill crop nutrient requirements. Corn stover is being sanctioned as an effective feedstock for the production of cellulosic bio-ethanol due to the higher cellulosic concentration, greater biomass production per unit area, and global availability^5–7^. Nonetheless, removing stover can decline soil quality, as well as agricultural productivity by reducing soil organic carbon (SOC) while enhancing the potential for soil degradation and erosion^8,9^. In contrast, incorporating corn stover into soil can improve SOC content, nutrient cycling, maintain soil structure, decrease soil erosion, and lead to improved microbial diversity. All of those parameters are contributing to soil quality, either directly or indirectly^6,9,10^. Soil microbes have an imperative role in soil processes and ecosystem utilities^10^. Hence, soil properties related to diversity, biomass and soil microbial community function can be used as soil quality indicators^11,12^ due to their fast response, ecological relevance, sensitivity and integrative qualities. Nevertheless, there are few studies reporting on the effects of stover management on soil enzymatic activity when coupled with fertilizer management. Soil enzymes can be used as potential indicators of soil quality because of their relationship to soil biology, ease of measurement, and rapid response to changes in soil management^13–15^. Such an index would integrate chemical, physical and biological characteristics and be used to monitor the effects of soil management on long-term productivity^16^. Enzymes catalyze all biochemical reactions and are an integral part of nutrient cycling in the soil^15^.

Research studies have reported that agricultural management practices have a significant impact on enzyme activities^13^. The relation between removal of stover to C returned to the system has to be considered to address economic concerns regarding increases in nutrient removal rates and replacement costs^6^ specially for nutrients such as N and P^17,18^. Fertilizer application is one of the largest expenses for farmers growing cereal crops and yet much of the N and P used to supplement crop needs are lost to the environment due to the low nutrient use efficiency of cereal crops^19–21^. Around 20–50% of N-based fertilizer can be lost to the environment as greenhouse gases (GHG) (*e.g.,* nitrous oxide, N_2_O), as well as, leaching and runoff (*e.g.,* nitrate, NO_3_-N)^22–25^. Phosphorus is the second most limiting nutrient in crop production after N. It is estimated that P deficiencies can be found in nearly 67% of world land designated for crop production^26^. In addition, P use efficiency in cereal crops in the world is low, varying between 15–30%^26^.

Over or under N and P fertilizer application can lead to a reduction in crop yield, in addition to creating conditions which favor nutrient losses to the environment, poor soil quality and plant nutrition^27^. Therefore, there is a need for improved nutrient management strategies, in particular N and P under different scenarios (*e.g.,* removed or incorporated residue management) to properly replace nutrients, ensuring adequate plant nutrition and at least sustained grain yield^20,28^. Sustainable agricultural production requires an inclusive framework that concurrently considers the impacts of production methods on soil health, including soil chemical and enzymatic activity. Therefore, we aimed to investigate the interactive effect of N and P fertilizers application under removed or incorporated residue management on soil nutrient availability and enzymatic activity in a continuous corn cropping system in the US Midwest. We hypothesized that stover maintenance would increase enzyme activities (β-glucosidase, alkaline phosphatase, arylsulfatase activities and fluorescein diacetate hydrolysis) compared to systems that stover was removed, leading to a greater inorganic N and P availability, reducing N and P-fertilizers dependance. This research could provide new clues on how residue management could influence soil health and N and P fertilization management, aiming at improved corn production sustainability and enhanced soil quality.

## Results

### Summary of analysis of variance

Supplemental Tables 1–3 presents the results from the analysis of variance. The following sections will focus on the main effects and interactions that were found to be significant at the *P*-level ≤ 0.05 as seen on Sup. Tables 1-3.

### Soil nutrient responses to residue management coupled with N and P fertilization

**Figure 1:**
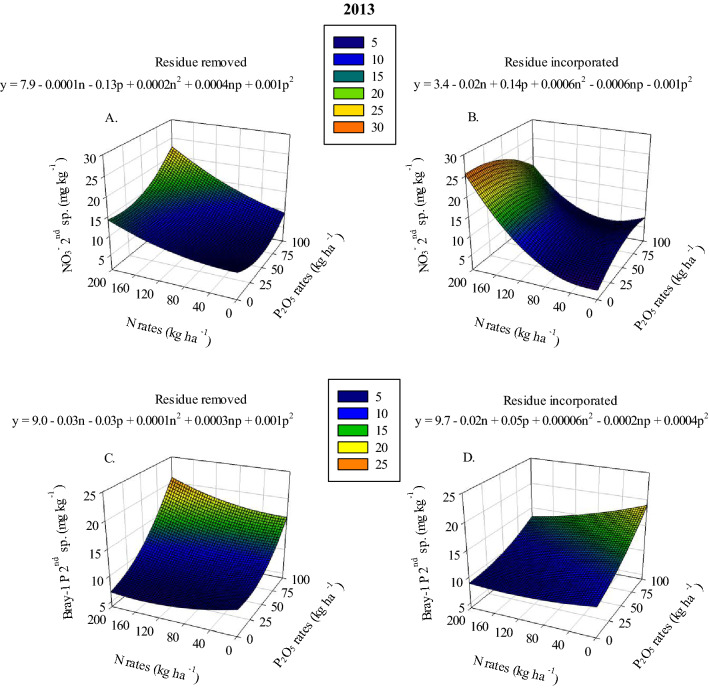
Nitrate (NO_3_^−^) under removed (**A**) and incorporated (**B**) residue management and Bray-1 P under removed (**C**) and incorporated (**D**) residue management in 2nd sampling in 2013 as a function of N and P_2_O_5_ rates.

**Figure 2:**
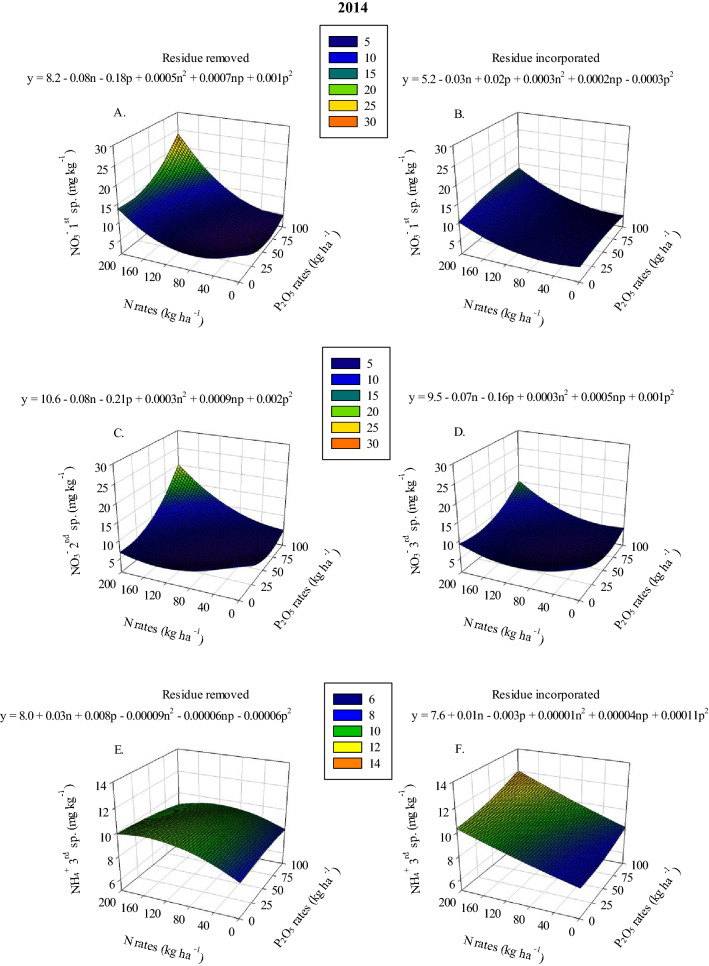
Nitrate (NO_3_^−^) under removed (**A**) and incorporated (**B**) residue management in 1st sampling, NO_3_^−^ in 2nd (**C**) and 3rd (**D**) samplings, ammonium (NH_4_^+^) under removed (**E**) and incorporated (**F**) residue management in 3rd sampling in 2014 as a function of N and P_2_O_5_ rates.

**Figure 3:**
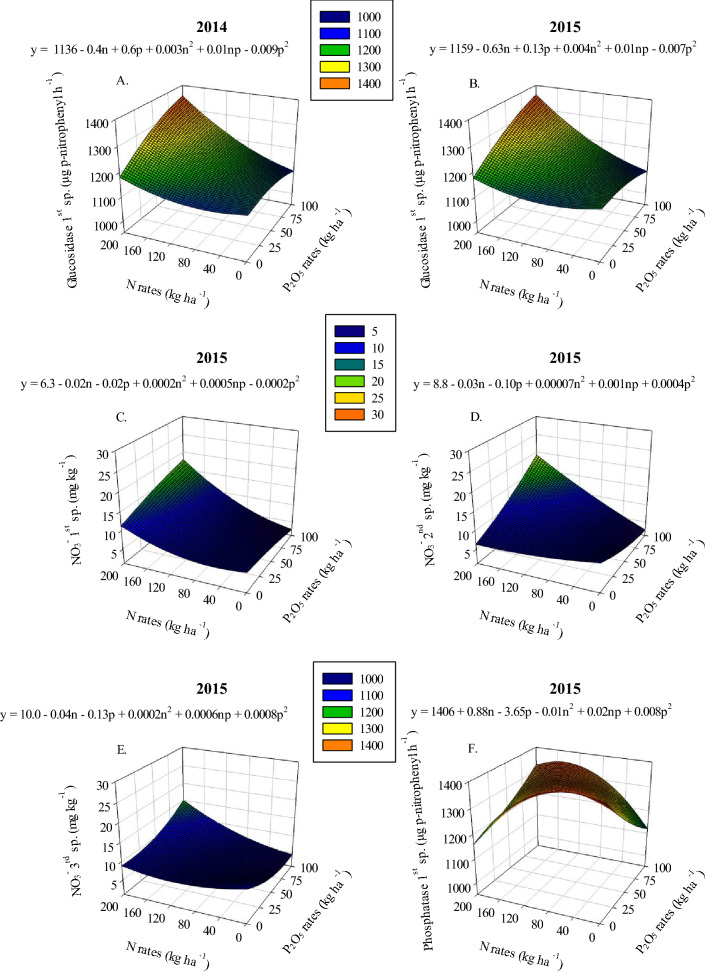
Glucosidase in 1st sampling in 2014 (**A**) and 2015 (**B**), nitrate (NO_3_^−^) in 1st (**C**), 2nd and 3rd (**D**) samplings in 2015 and phosphatase in 1st sampling in 2015 as a function of N and P_2_O_5_ rates. Glucosidase = β-Glucosidase; Phosphatase = Alkaline phosphatase.

Nitrate concentration response to increasing N and P_2_O_5_ application rates was found to vary based on residue management (Sup. Tables 1 and 2). In 2013 and 2014 (2nd and 1st samplings, respectively), when residue was removed the highest soil NO_3_^−^ concentration ranged between 22.6–23.7 mg kg^−1^ after the application of 160–200 kg N ha^−1^ combined with 75–100 kg P ha^−1^ (Figs. 1A and 2A). Maximum soil NO_3_^−^ concentration in plots where residue was incorporated, during the 2013 and 2014 years, ranged between 13.4–25.5 mg kg^−1^ at the highest N application rates combined with the lowest P_2_O_5_ application rates, 0–50 kg P ha^−1^ (Figs. 1B and 2B). This result shows that P has a significant effect on soil NO_3_^−^ concentration and the effect differs based on residue management. Residue management also has a significant effect on soil NO_3_^−^ concentrations, and NO_3_^−^ concentrations in plots where residue was removed were, on average, about 18% higher than where residue was incorporated. In the 2nd and 3rd samplings of 2014 and in all samplings of 2015, the highest soil NO_3_^−^ levels ranged between 14.9–20.0 mg kg^−1^ when 160–200 kg N ha^−1^ combined with 75–100 kg P_2_O_5_ ha^−1^ was applied regardless of residue management (Figs. 2C, 2D, 3C-3E).

**Figure 4:**
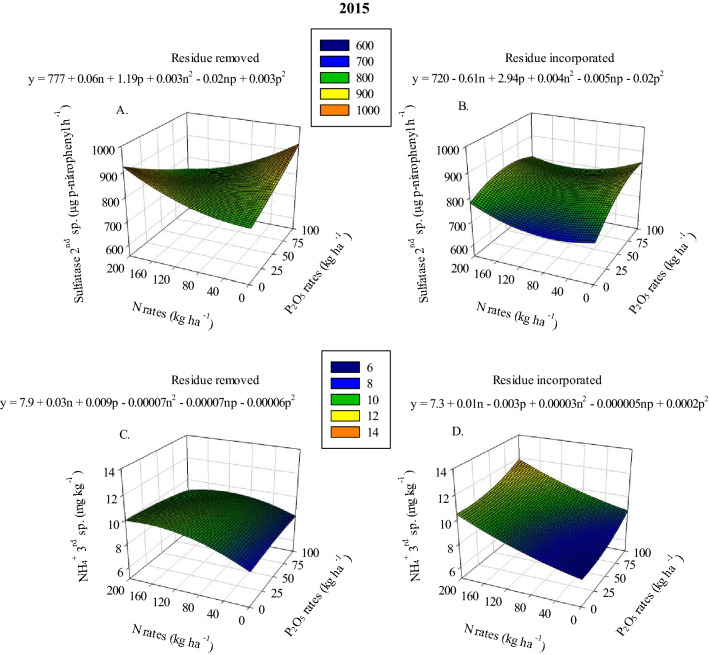
Sulfatase under removed (**A**) and incorporated (**B**) residue management in 2nd sampling, ammonium (NH_4_^+^) under removed (**C**) and incorporated (**D**) residue management in 3rd sampling in 2015 as a function of N and P_2_O_5_ rates. Sulfatase = Arylsulfatase.

Similarly, soil NH_4_^+^ concentration response to increasing N and P_2_O_5_ application rates varied based on residue management (Tables 1, 2 and 3). In the 1st sampling of 2013, soil NH_4_^+^ was 44.2% greater in plots where residue was incorporated compared to plots where residue was removed (Table 1). In 2014 and 2015 (3rd sampling for both years), when residue was removed, maximum soil NH_4_^+^ averaged 10.0 mg kg^−1^ after the application of 160–200 kg N ha^−1^ combined with 0–25 kg P_2_O_5_ ha^−1^ (Figs. 2E and 4C). While maximum NH_4_^+^ in plots where residue was incorporated, during the 2014 and 2015 seasons, averaged 12.0 mg kg^−1^ after the application of the highest N and P_2_O_5_ application rates (Figs. 2F and 4D). Higher P_2_O_5_ rates led to increased soil NH_4_^+^ concentration when residue was incorporated compared to where residue was removed. Furthermore, soil NH_4_^+^ in plots where residue was incorporated was, on average, about 20% higher than where residue was removed. In addition, in 2014 and 2015, soil NH_4_^+^ in plots where residue was removed had a higher rate of response to applied N (the increase in soil NH_4_^+^ per unit of N applied). In the 2nd sampling of 2014, the increase in soil NH_4_^+^ was found to have a rate of increase equivalent to 0.007 mg NH_4_^+^ kg^−1^ of applied N where residue was removed, which was significantly greater than where residue was incorporated, 0.005 mg NH_4_^+^ kg^−1^ (Table 2). Also, soil NH_4_^+^ concentration in the 1st sampling of 2015 showed greater rate of response to applied N in plots where residue was removed (6.7 mg NH_4_^+^ kg^−1^ applied N) compared with where residue was incorporated (5.1 mg NH_4_^+^ kg^−1^ applied N) (Table 2). However, when 167 kg N ha^−1^ was applied, the plots where residue was incorporated showed greater soil NH_4_^+^ compared to the plots where residue was removed residue (Table 2). These results regarding NO_3_^−^ and NH_4_^+^ concentrations suggest that there might be a link between stover management and the types of microbial activity taking place and how they impact the N cycle.

**Table 1:** Ammonium (NH_4_^+^), fluorescein diacetate hydrolysis (FDA) and glucosidase in 1st, 2nd and 3rd samplings in 2013, 2014 and 2015 as a function of residue management.

Source	**2013**	
	1st sampling	2nd sampling
	**NH**_**4**_^**+**^	**FDA**
	(mg kg^−1^)	(mg kg^−1^ in 3 h)
Residue removed	5.2 b ± 0.13†	425 b ± 8.8
Residue incorporated	7.5 a ± 0.27	550 a ± 14.6

Source	**2014**	
	3rd sampling	–
	**FDA**	–
	(mg kg^−1^ in 3 h)	–
Residue removed	572 b ± 19.2	–
Residue incorporated	793 a ± 15.2	–

Source	**2015**	
	2nd sampling	3rd sampling
	**Glucosidase**	**FDA**
	(µg p-nitrophenyl h^−1^)	(mg kg^−1^ in 3 h)
Residue removed	827 b ± 13.9	576 b ± 17.4
Residue incorporated	1055 a ± 19.2	793 a ± 15.4

^†^ Means within the column followed by different letters are significantly different (*p-value* ≤ *0.05*).

± Refers to the standard error of the mean.

Glucosidase = β-glucosidase.

**Table 2:** Nitrate (NO_3_^−^), ammonium (NH_4_^+^), Bray-1 P, phosphatase and glucosidase as a function of N rates and residue management.

Source	Residue management	Intercept	Slope lin	Slope quad	Maximum variable response
**2013**					
*1*st* sampling*					
NO_3_^−^ (mg kg^−1^)	-	3.37 ± 1.89	− 0.01x ± 0.03	0.0006x^2^ ± 0.0001	25 at 200 kg N ha^−1^
*2nd sampling*					
Glucosidase (µg p-nitrophenyl h^−1^)	Residue removed	952^b^ ± 35	0.68x ± 0.21	NS	1088^a^ at 200 kg N ha^−1^
Glucosidase (µg p-nitrophenyl h^−1^)	Residue incorporated	1055^a^ ± 34	NS	NS	1055^a^ at 200 kg N ha^−1^
**2014**					
*1st sampling*					
NH_4_^+^ (mg kg^−1^)	–	4.75 ± 0.16	0.006x ± 0.001	NS	6.0 at 200 kg N ha^−1^
Bray-1 P (mg kg^−1^)	–	31 ± 5.9	− 0.08x ± 0.03	0.0004x^2^ ± 0.0001	31 at 200 kg N ha^−1^
*2nd sampling*					
NH_4_^+^ (mg kg^−1^)	Residue removed	5.0^a^ ± 0.28	0.007^a^x ± 0.001	NS	6.4^a^ at 200 kg N ha^−1^
NH_4_^+^ (mg kg^−1^)	Residue incorporated	5.4^a^ ± 0.27	0.005^b^x ± 0.001	NS	6.4^a^ at 200 kg N ha^−1^
Glucosidase (µg p-nitrophenyl h^−1^)	–	909 ± 48	0.37x ± 0.11	NS	983 at 200 kg N ha^−1^
*3rd sampling*					
Bray-1 P (mg kg^−1^)	–	24 ± 4.45	-0.06x ± 0.03	0.0003x^2^ ± 0.0001	24 at 200 kg N ha^−1^
**2015**					
*1*st* sampling*					
NH_4_^+^ (mg kg^−1^)	Residue removed	4.17^b^ ± 0.29	0.03x ± 0.007	− 0.00009x^2^ ± 0.00003	6.7^a^ at 167 kg N ha^−1^
NH_4_^+^ (mg kg^−1^)	Residue incorporated	5.1^a^ ± 0.29	NS	NS	5.1^b^ at 167 kg N ha^−1^
Phosphatase (µg p-nitrophenyl h^−1^)	Residue removed	1579^a^ ± 358	-2.02x ± 0.84	0.01x^2^ ± 0.004	1579^a^ at 0 kg N ha^−1^
Phosphatase (µg p-nitrophenyl h^−1^)	Residue incorporated	1713^a^ ± 360	NS	NS	1713^a^ at 0 kg N ha^−1^
*2*nd* sampling*					
NH_4_^+^ (mg kg^−1^)	–	5.3 ± 0.16	0.005x ± 0.0007	NS	6.3 at 200 kg N ha^−1^
Glucosidase (µg p-nitrophenyl h^−1^)	–	913 ± 41	0.31x ± 0.11	NS	975 at 200 kg N ha^−1^

NS = not significant.

^†^ Means within the column followed by different letters are significantly different (*p-value* ≤ *0.05*).

± Refers to the standard error of the mean.

Glucosidase = β-glucosidase; Phosphatase = Alkaline phosphatase.

**Table 3:** Ammonium (NH_4_^+^), Bray-1 P, sulfatase, glucosidase and fluorescein diacetate hydrolysis (FDA) as a function of P_2_O_5_ rates and residue management.

Source	Residue management	Intercept	Slope lin	Slope quad	Maximum variable response
**2013**					
2nd* sampling*					
NH_4_^+^ (mg kg^−1^)	-	15.09 ± 2.03	-0.07x ± 0.05	0.001x^2^ ± 0.0005	18.1 at 100 kg P_2_O_5_ ha^−1^
**2014**					
*1*st* sampling*					
Bray-1 P (mg kg^−1^)	-	23 ± 6.9	0.13x ± 0.01	NS	36 at 100 kg P_2_O_5_ ha^−1^
Sulfatase (µg p-nitrophenyl h^−1^)	Residue removed	1188^a^ ± 189	NS	NS	1188^a^
Sulfatase (µg p-nitrophenyl h^−1^)	Residue incorporated	1140^a^ ± 191	NS	NS	1140^a^
*2*nd* sampling*					
Glucosidase (µg p-nitrophenyl h^−1^)	Residue removed	871^b^ ± 44	-2.75x ± 1.17	0.03x^2^ ± 0.01	896^b^ at 100 kg P_2_O_5_ ha^−1^
Glucosidase (µg p-nitrophenyl h^−1^)	Residue incorporated	1050^a^ ± 44	NS	NS	1050^a^ at 100 kg P_2_O_5_ ha^−1^
*3*rd* sampling*					
Sulfatase (µg p-nitrophenyl h^−1^)	Residue removed	1101^a^ ± 125	1.25^a^x ± 0.58	NS	1226^a^ at 100 kg P_2_O_5_ ha^−1^
Sulfatase (µg p-nitrophenyl h^−1^)	Residue incorporated	1106^a^ ± 126	-1.36^b^x ± 0.51	NS	1106^a^ at 100 kg P_2_O_5_ ha^−1^
**2015**					
*1*st* sampling*					
Bray-1 P (mg kg^−1^)	–	19.6 ± 5.9	0.26x ± 0.04	-0.002x^2^ ± 0.0004	28 at 65 kg P_2_O_5_ ha^−1^
*2*nd* sampling*					
Bray-1 P (mg kg^−1^)	–	16.4 ± 4.4	0.16x ± 0.01	NS	32 at 100 kg P_2_O_5_ ha^−1^
*3*rd* sampling*					
Bray-1 P (mg kg^−1^)	Residue removed	13.9^a^ ± 5.8	NS	NS	13.9^b^ at 100 kg P_2_O_5_ ha^−1^
Bray-1 P (mg kg^−1^)	Residue incorporated	16.7^a^ ± 5.8	0.19x ± 0.06	NS	36^a^ at 100 kg P_2_O_5_ ha^−1^
Sulfatase (µg p-nitrophenyl h^−1^)	Residue removed	1092^a^ ± 125	1.34^a^x ± 0.48	NS	1226^a^ at 100 kg P_2_O_5_ ha^−1^
Sulfatase (µg p-nitrophenyl h^−1^)	Residue incorporated	1086^a^ ± 124	-1.15^b^x ± 0.44	NS	1086^a^ at 100 kg P_2_O_5_ ha^−1^
FDA (mg kg^−1^ in 3 h)	-	648 ± 32	2.58x ± 0.78	− 0.03x^2^ ± 0.007	713 at 43 kg P_2_O_5_ ha^−1^

NS = not significant.

^†^ Means within the column followed by different letters are significantly different (*p-value* ≤ *0.05*).

± Refers to the standard error of the mean.

Glucosidase = β-glucosidase; Sulfatase = Arylsulfatase.

For soil Bray-1 P levels during the 2013 season, a response to applied P_2_O_5_ and N was observed only in the 2nd sampling. It was observed that the highest soil Bray-1 P levels were observed when residue was removed, and it ranged between 19.3–20.9 mg kg^−1^ after the application of 160–200 kg N ha^−1^ combined with 75–100 kg P ha^−1^ (Fig. 1C). Maximum Bray-1 P in plots where residue was incorporated, during the 2013 2nd sampling, ranged between 17.4–18.9 mg kg^−1^ with the highest P application rates but lower N application rates, 0–40 kg N ha^−1^ (Fig. 1D). This result shows that lower N rates led to higher soil Bray-1 P when residue was incorporated compared with plots where residue was removed. However, soil Bray-1 P in plots where residue was incorporated was, on average, about 11% lower than plots where residue was removed. Soil Bray-1 P response to increasing N and P_2_O_5_ application rates was found to be greater when the highest N rates were applied in the 2014 and 2015 seasons (Tables 2 and 3). In the 3rd sampling of 2015, higher soil Bray-1 P was observed in plots where residue was incorporated (36 mg kg^−1^ at 100 kg P_2_O_5_ ha^−1^) than in plots where residue was removed (19 mg kg^−1^ at 100 kg P_2_O_5_ ha^−1^) (Table 3).

### Soil enzymatic activity responses to residue management coupled with N and P fertilization

Soil phosphatase activity showed very few responses to applied N and P. The most significant result observed was that soil phosphatase activity ranged between 1318–1351 µg p-nitrophenyl h^−1^ when 120–160 kg N ha^−1^ was applied combined with 75–100 kg P_2_O_5_ ha^−1^ in the 1st sampling of 2015 (Fig. 3F). Soil sulfatase activity response to increasing P_2_O_5_ application rates was found to vary based on residue management (Table 3). In the 3rd sampling of the 2014 and 2015 growing season, soil sulfatase activity increased linearly with increasing P_2_O_5_ application rates when residue was removed; in contrast, soil sulfatase activity decreased linearly with increasing P_2_O_5_ application rates when residue was incorporated (Table 3). In addition, soil sulfatase activity in plots where residue was removed had a higher rate of response to P_2_O_5_ application rate, as indicated by the significantly greater linear slope (Table 3). Soil sulfatase activity was also affected by N application (Sup. Table 3). In the 2nd sampling of 2015, when residue was removed the greatest soil sulfatase activity ranged between 872–929 µg p-nitrophenyl h^−1^ and was observed after the application of 160–200 kg N ha^−1^ combined with 0–25 kg P_2_O_5_ ha^−1^ or 0–40 kg N ha^−1^ combined with 75–100 kg P_2_O_5_ ha^−1^ (Fig. 4A). The greatest soil sulfatase activity in plots where residue was incorporated ranged between 850–854 µg p-nitrophenyl h^−1^ and was observed after the application of 0–40 kg N ha^−1^ combined with 75–100 kg P_2_O_5_ ha^−1^ (Fig. 4B).

Soil glucosidase activity was found to be greatest when the highest rates of N and P_2_O_5_ were applied at the 1st sampling of 2014 and 2015 (Figs. 3A and 3B). The highest soil glucosidase activity ranged between 1328–1360 µg p-nitrophenyl h^−1^ with the application of 160–200 kg N ha^−1^ combined with 75–100 kg P_2_O_5_ ha^−1^ for both years (Figs. 3A and 3B). Soil glucosidase activity also responded linearly to increasing N application rates in the 2nd sampling of 2014 and 2015 (Table 2). However, during the 2nd sampling in the 2013 and 2014 season, soil glucosidase activity response to N and P_2_O_5_ rates varied based on residue management (Tables 2 and 3). In the 2nd sampling of 2013, soil glucosidase activity showed a positive linear response to applied N when residue was removed, with no trends observed when residue was incorporated (Table 2). In the 2nd sampling of 2014, when residue was removed, soil glucosidase activity responded non-linearly to increasing P_2_O_5_ application rates, with no trends observed when residue was incorporated (Table 3). In addition, in the 2nd sampling of 2015, soil glucosidase activity was 27.6% greater in plots where residue was incorporated compared to plots where residue was removed (Table 1).

Fluorescein diacetate hydrolysis was greater in plots where residue was incorporated compared to plots where residue was removed in the 2nd sampling of 2013 (an increase of 29.4%) and 3rd sampling of 2014 and 2015 (an increase of 38.6% and 37.7%, respectively) (Table 1). In addition, FDA was found to respond non-linearly to increasing P_2_O_5_ application rates with maximum value of 713 mg kg^−1^ in 3 h^−1^ at 43 kg P ha^−1^ (Table 3).

### Principal component analysis

The eigenvalues of the four extracted principal components were greater than 1 and these components can, therefore, be grouped into a four‐component model which accounts for 78% and 75% of data variation in the plots where residue was removed and incorporated, respectively (Table 4).

**Table 4:** Factor loadings of a principal component analysis; bold loadings > 0.3.

Parameters	PC1	PC2	PC3	PC4
**Residue removed**				
NO_3_^−^	0.085	**0.387**	**0.824**	− 0.200
NH_4_^+^	0.047	− 0.207	**0.359**	**0.891**
Bray-1 P	**0.547**	0.135	0.109	− 0.113
Phosphatase	**0.543**	0.096	**− 0.330**	0.178
Sulfatase	**− 0.510**	**0.419**	− 0.086	0.058
Glucosidase	0.182	**0.743**	− 0.230	0.247
FDA	**0.319**	− 0.223	0.093	− 0.235
Variance (%)	31	17.9	14.6	14.1
Cumulative variance (%)	31	49	64	78
Eingenvalues	2.204	1.253	1.022	1.000
**Residue incorporated**				
NO_3_^−^	− 0.175	-0.294	**0.683**	0.220
NH_4_^+^	0.245	**-0.576**	**0.386**	− 0.006
Bray-1 P	0.267	**0.314**	0.196	**− 0.402**
Phosphatase	**0.623**	0.206	0.067	0.176
Sulfatase	**− 0.642**	0.066	− 0.044	0.087
Glucosidase	− 0.077	**0.634**	**0.449**	**0.440**
FDA	0.171	− 0.179	−	**0.745**
Variance (%)	28	16.5	15.8	14.0
Cumulative variance (%)	28	45	61	75
Eingenvalues	1.981	1.153	1.107	1.000

FDA = fluorescein diacetate hydrolysis.

For plots where residue was removed, PC1 represented 31% of the variance and showed soil Bray-1 P, phosphatase activity and FDA as positively concordant (Table 4). Conversely, soil sulfatase activity was negatively correlated with the PC1 components (Table 4). Principal component 2 showed that soil sulfatase and glucosidase activity increased as NO_3_^−^ concentration in the soil increased (Table 4). Principal component 1 and PC2 represented 49% of the cumulative variance (Table 4). The other two extracted factors are of minor importance in terms of both eigenvalues and explained variability (Table 4). Principal component 3 showed a positive correlation between soil NO_3_^−^ and NH_4_^+^ concentration, however, a negative correlation between soil inorganic nitrogen and soil phosphatase activity (Table 4).

For plots where residue was incorporated, PC1 represented 28% of the variance and showed negative correlation between soil phosphatase and sulfatase activity (Table 4). Principal component 2 showed that soil glucosidase activity increased as soil Bray-1 P concentration increased; however, soil NH_4_^+^ concentration was negatively correlated with both soil glucosidase activity and Bray-1 P (Table 4). Principal component 1 and PC2 represented 45% of the cumulative variance (Table 4). Similarly, as observed in plots where residue was removed, the other two extracted factors are of minor importance in terms of both eigenvalues and explained variability (Table 4). Principal component 3 showed that soil NO_3_^−^ and NH_4_^+^ concentration and glucosidase activity as positively concordant (Table 4). In contrast, FDA was negatively correlated with all the previously reported parameters (Table 4). Principal component 4 showed that soil glucosidase activity and FDA decreased as soil Bray-1 P concentration increased (Table 4).

**Figure 5:**
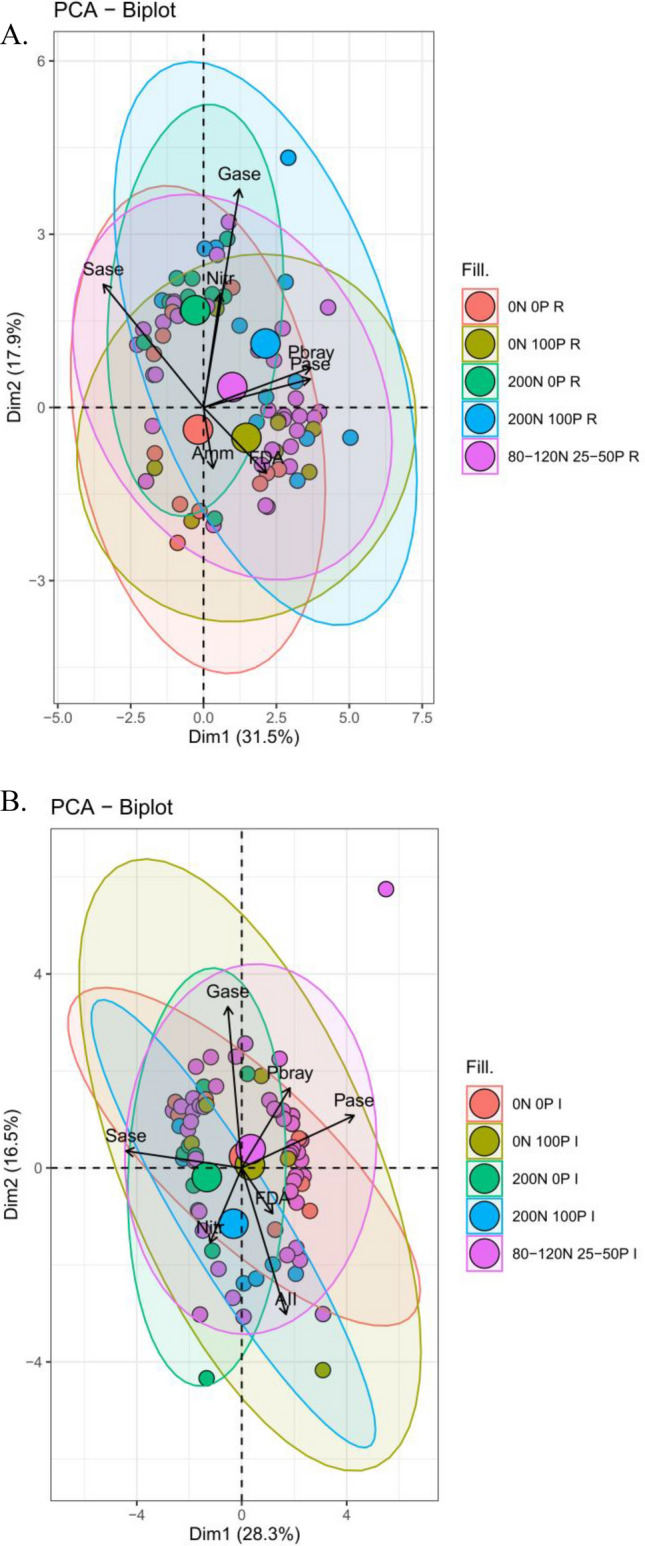
Biplot graphics of principal component analysis among the relationship between nitrate (NO_3_^−^), ammonium (NH_4_^+^), Bray-1 P (Pbray), phosphatase (Pase), sulfatase (Sase), glucosidase (Gase) and fluorescein diacetate hydrolysis (FDA) evaluated in residue removed management (A) and residue incorporated management (B). 0 N 0P = Absence of N and P fertilization; 0 N 100P = Absence of N and application of 100 kg P_2_O_5_ ha^−1^; 200 N 0P = Application of 200 kg N ha^−1^ and absence of P fertilization; 200 N 100P = Application of 200 kg N ha^−1^ and 100 kg P_2_O_5_ ha^−1^; 80-120 N 25-50P = Application of 80–120 kg N ha^−1^ and 25–50 kg P_2_O_5_ ha^−1^. R and I refers to removed and incorporated residue management.

Analyzing the grouped PCA biplot graph (PC1 and PC2), under the conditions where residue was removed shows that the group formed by the highest N and P_2_O_5_ application rates (200 kg N ha^−1^ and 100 kg P_2_O_5_ ha^−1^) best comprised most of soil chemical and enzymatic analysis (Fig. 5A). Whereas, plots where residue was incorporated, the group formed by lower N and P_2_O_5_ application rates (80–120 kg N ha^−1^ and 25–50 kg P_2_O_5_ ha^−1^) was found to relate better to the soil chemical and enzymatic analysis performed (Fig. 5B).

## Discussion

### Inorganic N and P availability responses to N and P-fertilization under different stover management

Bray-1 P and soil phosphatase activity were not greatly affected by corn stover removal, except in the 2nd sampling of 2013. Initial Bray-1 P content in soil (11 mg kg^−1^) was within the medium range (11–15 mg kg^−1^) according to fertilizer guidelines for agronomic crops in Minnesota^29^, therefore, slight response to residue management should be expected. In addition, soil available P probably exceeded microbial demand after P addition. The most likely mechanism for this is that P input provides an extra source of inorganic P, thus reducing the microbial dependence on organic P mineralization as the main source of P^30^. Microbes can reduce metabolic function related to phosphatase enzyme production when sufficient inorganic P is available^31^. This mechanism is commonly referred to as “end-product suppression” and found frequently in soils where available P is added^30^.

In contrast, N application had a significant impact on P availability based on how residue was managed. Plots where residue was incorporated required lower N application rates to achieve maximum P availability compared with plots where the residue was removed. In addition, P application also played a significant role in the behavior of soil NO_3_^−^ and NH_4_^+^ based on residue management. It was observed that soil NO_3_^−^ availability where residue was incorporated needed lower P_2_O_5_ application rates to achieve maximum levels compared with plots where the residue was removed. In contrast, soil NH_4_^+^ levels where residue was incorporated needed higher P_2_O_5_ application rates to achieve maximum levels compared with plots where the residue was removed. Nitrogen and P are the two most abundant mineral nutrients for plants, and their coordinated acquisition is vital for plants to achieve nutritional balance and optimal growth under a fluctuating nutritional environment^32^. Nitrate and phosphate are the major sources of N and P for plants, and they also act as the signal molecules to trigger downstream N or P responses^33^. Thus, the uptake of N and/or P affect each other, indicating the strategy that has evolved for maintaining N–P balance in soil^33^ as verified in our study.

Soil NH_4_^+^ response to N application rates was less evident under plots where residue was incorporated compared with plots where residue was removed. In addition, in the 1st sampling of 2013 NH_4_^+^ was 44.2% greater in plots where the residue was incorporated compared with plots where residue was removed. In general, NO_3_^−^ in plots where residue was incorporated was, on average, about 18% lower than where residue was removed. In contrast, NH_4_^+^ in plots where residue was incorporated was, on average, about 20% higher than where residue was removed. This result indicates that residue management also plays a role on the relation between soil NO_3_^−^ and NH_4_^+^ availability. The balance among N transformations largely determines whether N is retained or lost from soils^34–36^. Under field conditions, NH_4_^+^ existing in soil, no matter whether it comes from N-fertilization or from organic matter (OM) mineralization, can swiftly transformed into NO_3_^−^ through nitrification^34,37^. Nitrification and denitrification are well known important processes for N losses via nitrous oxide (N_2_O) emissions and NO_3_^−^ leaching in agricultural systems^36,38^. The dynamics of the biological N cycle in cultivated soils could be resumed with three main processes^38,39^: (i) Decomposition of soil microorganisms and plant litter to OM, which can be reduced to NH_4_^+^-N and dissolved organic N; (ii) assimilative processes of NO_3_^–^N and dissolved organic N, by microorganisms and plants for replication and growth; and (iii) other processes including dissimilatory NO_3_^−^ reduction to NH_4_^+^, nitrification, denitrification and biological N fixation (BNF), as well as newly described pathways such as anammox and codenitrification^38^. Thus, the stover management as a substrate supply could be related to controlling nitrification in cultivated soils. The availability of O_2_ , NO_2_^−^ and NH_4_^+^ often shapes both the size of the resultant nitrifier populations and the rate of nitrification^40–43^. Under cropping systems environments, the substrate pool of NH_4_^+^ is increased by the addition of urea and ammoniacal fertilizers, NH_4_^+^ production via mineralization, atmospheric deposition of NH_4_^+^, deposition of animal wastes, and BNF^43^. In contrast, the competing consumptive processes including microbial assimilation (immobilization), plant assimilation, and NH_3_ volatilization decrease available NH_4_^+^^43^.

Sources of soil carbon (C) and OM such as crop residues (specifically corn stover in this study) has been widely promoted in agroecosystems all over the world to increase soil C stock, improving synchrony of nutrients supply (*e.g.,* from mineral fertilization)^44–46^. Therefore, keeping crop residues in the field could reduce the amount of N that leaches during the initial stage of crop growth when N demand is low, especially when there is high soil N leaching risk (*e.g.,* the snow melting or heavy spring rain events during early crop growth stages)^6,46–49^. The subsequent remineralization of the immobilized N can supply mineral N for crop growth, indicating that the incorporation of corn stover, which contains a larger amount of C with high availability, can improve N synchrony to corn cropping systems by increasing NH_4_^+^ availability. However, increasing the amount of incorporated residue may intensify N immobilization, which may augment the requirements to additional N fertilizer application over the time^50,51^.

### Enzymatic responses to N and P-fertilization under different stover management

Positively charged NH_4_^+^ is easier absorbed by negatively charged soil colloids than NO_3_^−^; as a result, NH_4_^+^ stays in the soil longer and is more available to microorganisms than NO_3_^–34^. Yan et al.^52^ verified that increased NH_4_^+^ content in soil promoted greater activities of β-1,4-glucosidase and β-1,4-N-acetylglucosaminidase in soil aggregates than increased soil NO_3_^−^. Thus, higher levels of NH_4_^+^ in soils provided by residue incorporation could impact soil enzymatic activity. Microbial biomass and enzyme activity are very sensitive indicators of the variation of C flux. It is well known that SOC, microbial biomass and enzyme activity are correlated^13,53,54^. In particular, soil microbial biomass and activity, and enzyme activities have been shown to be more sensitive than total organic C to soil disturbance resulting from residue management^54–56^. This means that increase/reduction of these parameters directly reflects an increase/reduction of C input to soil. The incorporation of C-based organic material to soil help in maintaining SOC levels, which in turns typically improves the moisture retention, nutrient status, aeration, nutrient supply, and biological function of soils^57,58^. Glucosidase enzymes are well known for their critical role in releasing low molecular weight sugars that are important as energy sources for microorganisms related to the C cycle, acting in the cleavage of cellobiose into glucose molecules^15^. Thus, the effect of soil NH_4_^+^ on glucosidase activity is likely to lead to an increased cellulose (mainly from crop residues) input to soil which, in turn, was a result of the increased plant biomass production resulting from NH_4_^+^ application. Therefore, there is an indirect positive effect of NH_4_^+^ on β -glucosidase.

Our results also showed that soil sulfatase and glucosidase activity response to N and P_2_O_5_ application in plots where residue was incorporated was lower compared with plots where the residue was removed. In addition, corn stover incorporation provided greater soil glucosidase activity in the 2nd sampling of 2015 (an increase of 27.6%) and FDA in the 2nd sampling of 2013 (an increase of 29.4%) and 3rd sampling of 2014 and 2015 (an increase of 38.6% and 37.7%, respectively). Residue removal has previously been shown to reduce soil microbial activities^59–61^. Our data indicate that FDA was the biological parameter most affected by stover removal, followed by glucosidase. Fluorescein diacetate can be hydrolyzed by many enzymes (lipases, proteses and esterases) and organisms, being considered a broad-spectrum indicator of soil biological activity^15^. Although arylsulfatase response to stover management was less evident, this enzyme is important in nutrient cycling because it releases plant available SO_4_. Also, it may be an indirect indicator of fungi as only fungi (not bacteria) contain ester sulfate, the substrate of arylsulfatase^13^. Furthermore, the application of 10 kg ha^−1^ of sulfur as potassium sulfate and potassium chloride in the entire experimental site would impair arylsulfatase activity.

The fact that the response to N and P_2_O_5_ application rates were less evident when residue was incorporated suggests that the removal of corn stover may deplete soil organic C (SOC) and have a potential negative impact on nutrient availability, in addition, to reducing microbial enzymatic activity. One of the most important considerations related to crop residue management is the effect on soil quality parameters such as SOC, soil organic matter (SOM) soil pH, nutrient balances, water holding capacity and aggregate stability that would affect soil microbes and plants development^62–66^. Corn residues protect soils from the erosive forces of water and wind, maintain SOC stocks, cycle essential plant nutrients, replenish the C that creates and sustains aggregation, and provide food and energy for the microbial community^67–69^. The availability of organic C is a well-known factor limiting the activity of heterotrophic microorganisms. A decreased biomass and microbial activity can lead to an alteration of soil quality, as microbial communities play a key role in essential ecosystem services and soil functions^10,70^. Kushwaha et al.^71^ reported that the activity and biomass of soil microbes were boosted with C from the incorporation of crop residues. However, Halpern et al.^72^ described in a long-term 16 years study that values of soil microbial biomass and activity are not always clearly reflected in the SOC pool. The removal of crops residue has been reported to also remove nutrients from the field^73^. The quantity of N, P (as P_2_O_5_) and K (as K_2_O) removed from field when residue is harvested has been estimated to range between 5.2–8.8, 0.6–3.1 and 7.2–20 kg Mg^−1^ of residue removed, respectively^73–75^. Therefore, to maintain optimum soil fertility nutrients must be replaced by fertilizer application, as suggested in the present study and also by Sawyer et al.^76^.

Although soil chemical and enzymatic activity showed different responses to N and P_2_O_5_ application rates in plots where residue was either removed or incorporated, in general, the highest nutrient availability and enzymatic activity were observed when the highest N and P_2_O_5_ rates were applied, mainly in 2014 and 2015. N-fertilizer application may shift soil microbial community structure and functions in different ways^77^. Microorganisms will allocate energy to the relatively absent resources so that N additions will cause C and P-acquisition enzymes to increase, and N-acquisition enzymes to decrease^34,78^. It has been reported that, when inorganic N forms were not considered, N additions caused C-degradation enzymes (α-1,4-glucosidase, β-1,4-glucosidase, cellobiohydrolase and β-1,4-xylosidase) and P-degradation enzymes (acid and alkaline phosphatases) to increase and restricted oxidase enzymes (polyphenol oxidase and peroxidase). However, N additions did not inhibit N-degradation enzymes (β-1,4-N-acetylglucosaminidase)^34,79,80^. The growth of soil microorganisms can be stimulated by improving soil N availability^77,81^. For example, De Deyn et al.^82^ related that N addition influenced microbial community structure by directly enhancing soil N availability, as well as by indirectly affecting soil microbial functions related to C turnover. Also, according to Li et al.^83^, it appears that P fertilization has fewer effects than N fertilization on soil microbial communities. These findings could explain the highest observed nutrient availability and enzymatic activity when the highest N rate was applied in combination with the highest P_2_O_5_ rate.

The PCA analysis showed that residue incorporation could be used to lower the amount of N and P_2_O_5_ needed for optimum soil levels. This does not contradict previously reported results since it merely showed that although the maintenance of residues in cropping systems have several benefits, for example, improving soil chemical, physical and biological properties^84^, it is not enough to completely supply macro and micronutrients demands for proper corn development. Removing an excessive amount of corn stover can result in soil degradation^59,85,86^. However, without stover harvest, producers can encounter residue management problems with subsequent crops and therefore often increase their tillage intensity to reduce surface residues^87–90^. Residue management is essential to balancing soil health and with long-term cropland productivity. In cropping systems, stover removal for biofuel production or other uses needs to be managed carefully to preserve the soil resource including SOC stocks ^2,6,90,91^. Therefore, new studies regarding partial stover removal should be performed. For example, Blanco-Canqui and Lal^8^ reported that 25% of corn stover could be removed from the field without negatively affecting soil fertility, SOC and structural stability. Differently, some authors suggests that 40% removal by mass was an upper limit for maintaining SOC and preventing erosion^67,69^. Johnson et al.^92^ concluded that 30 to 70% residue cover are required to sustain adequate SOC. Hence, assumptions only can be applied considering the specific soil-environment conditions for each agricultural system.

## Conclusions

This is the first study conducted in MN to report what the effects of stover management and fertilizer application are on selected soil biochemical properties. We have now showed that the availability of nutrients and enzyme activities in soils varies based on how residue is managed and also the amounts of N and P that are applied. Our results showed that in general, corn stover management significantly impacted soil mineral-N forms and enzymatic activity. It was observed that plots where residue was incorporated were found to have high NH_4_^+^ and enzymatic activity compared to plots where residue was removed. In contrast, plots where residue was removed showed higher NO_3_^−^ than plots where residue was incorporated. Nutrient availability and enzymatic activity were also found to be affected by residue management, in most cases, these parameters frequently showed a positive response to added N and P when residue was removed; whereas the response to applied N and P in plots where residue was incorporated was less evident. In general, lower nutrient availability was verified from plots where residue was removed than from plots where residue was incorporated, showing potential for nutrient deficiency if nutrients are not replaced accordingly. Careful consideration over soil available nutrients is of utmost importance in conditions where residue is being removed for energy production, use as bedding, or any other use. Furthermore, residue removal can decrease the activity of different enzymes related to C-cycle (β-glucosidase) and soil microbial activity (fluorescein diacetate hydrolysis—FDA) over continuous cropping seasons, impairing soil health.

## Materials and methods

### Site description and experimental design

This study was conducted under field conditions from 2013 to 2015 in Lamberton, state of Minnesota, United States (44°13′N and 16°01′W, 348 m above sea level (a.s.l.)). The maximum and minimum monthly temperatures and rainfall observed during the field trial are presented in Sup. Figure 1. The crop sequence used prior to the start of this experiment was soybean [*Glycine max* L. (Merril)] in 2011 followed by corn in 2012. A continuous corn system was used from 2013 to 2015. The soil was classified as a Canisteo clay loam (fine-loamy, mixed, superactive, calcareous, mesic Typic Endoaquolls). In the fall of 2012, 256 soil samples from 0 to 0.20 m were collected from the entire experimental area on sub plots of 205 m^2^ and were used for baseline determination of soil fertility. After collection, soils were air-dried, sieved (2 mm), and stored at room temperature (22° C) until analyses. Soil pH was measured in water (1:1 ratio w/v) and was 7.2 and organic matter was measure after loss on ignition and was 46.5 g kg^−1^. Soil test P was extracted with the Bray-1 reagent (average of 11 mg kg^−1^)^93^ and determined by the molybdate blue method of Murphy and Riley^94^ using a Biotek Epoch microplate spectrophotometer (Biotek, Winooski, VT). Ammonium and nitrate were analyzed according Nelson^95^ and Gelderman and Beegle^96^ after extraction in 2 M KCl using the sodium salicylate method (NH_4_^+^-N average of 4.8 mg kg^−1^) and vanadium method (NO_3_^–^N average 5.0 mg kg^−1^).

The study was set up in a full factorial (6 N rates × 5 P_2_O_5_ rates × 2 residue management) completely randomized design with four replications. Because of the size of the land area used for this research the experimental area was divided into two sections a north section and a south section. Each section was composed of six main blocks and each block consisted of 90 3 m x 12 m plots. Each experimental plot consisted of four 0.76 m spaced corn rows. The same plots received the same fertilizer treatments every year.

The treatments included six N application rates, five P_2_O_5_ application rates, and two residue management strategies. Nitrogen application rates ranged from 0 to 200 kg N ha^−1^ in 40 kg increments (0, 40, 80, 120, 160 and 200 kg N ha^−1^); P application rates ranged from 0 to 100 kg P_2_O_5_ ha^−1^ in 25 kg increments (0, 25, 50, 75 and 100 kg P_2_O_5_ ha^−1^). Nitrogen was applied as urea (46% N) and P as triple superphosphate (46% P_2_O_5_). Sulfur (10 kg ha^−1^) and K_2_O (70 kg ha^−1^) were also applied to assure no other nutrients were causing deficiency as potassium sulfate and potassium chloride following the University of Minnesota guidelines^29^. All fertilizer treatments were hand applied to each individual plot in the spring and incorporated with tillage immediately after application and prior to planting.

The two residue management treatments were: residue removed in the fall after harvest (as much as possible being removed by baling, about 90–95% residue was removed) or residue maintained (100% of the residue was incorporated into the soil). Tillage operations consisted of disk ripping to a depth of 25 cm in the fall after corn harvest, and field cultivating to a depth of 9 cm in the spring prior to planting corn in all plots regardless of residue management. Corn seeds were planted at 86,487 seeds ha^−1^, weeds were controlled using pre- and post-emergence herbicides, and insects were controlled using best management practices.

### Sample collection and analysis

Soil samples were randomly collected from 0–0.20 m depth within each plot by using a metal soil probe (2.5 cm diameter). Ten cores were collected from each plot and combined into one composite sample. There were three soil sampling times performed in each crop year (2013 to 2015): i) when the corn was at the V6 stage (6 leaves completely unfolded), ii) when the corn was at the R1 stage (female flowering) and iii) after corn harvest. In 2013, the samplings at V6 stage were not performed due to harsh environmental conditions (heavy rainfall and flooding). Thus, we decided to maintain the samplings at R1 stage and after corn harvest.

Soil samples were refrigerated at 4 °C and transported to the Southwest Research and Outreach Center, Minnesota, USA. As we mentioned before, soil NH_4_^+^ and NO_3_^−^ were determined according Nelson^95^ and Gelderman and Beegle^96^ and P was determined using the Bray-1 extractant^93^. Alkaline phosphatase (E.C. 3.1.3.1), arylsulfatase (E.C. 3.1.6.1) and β-glucosidase (E.C. 3.2.1.21) activities were determined according to Tabatabai^97^. Fluorescein diacetate hydrolysis (FDA) was analyzed based on adapted methodology from Adam and Duncan^98^. For all enzymes, the reactions were measured compared to a control from the same soil sample to account for p-nitrophenol released from activity not related to enzymes. In addition, blank solutions without soil, and blank solutions without reagents were used as quality control.

### Statistical analysis

Data were analyzed at *P* ≤ 0.05 using the mixed procedure of SAS 9.4^99^. The main effects included in the models were N and P application rate, sampling time, residue management, year, and their interactions. Nitrogen and P application rates were considered continuous variables and therefore required the use of regression analysis; residue management and year were considered fixed effects, and sampling time was considered a repeated measurement. The covariance structure that best fit the model for each parameter was assessed by checking the Akaike Information Criteria (AIC) among all possible covariance structures. Pairwise mean comparisons were made at *P* ≤ 0.05.

Principal component analysis (PCA) was used to assess the chemical and enzymatic activity of the soil. The following treatments were selected to represent the entire dataset: 0 N 0P = absence of N and P fertilization; 0 N 100P = absence of N and application of 100 kg P ha^−1^; 200 N 0P = application of 200 kg N ha^−1^ and absence of P fertilization; 200 N 100P = application of 200 kg N ha^−1^ and 100 kg P ha^−1^; 80-120 N 25-50P = application of 80–120 kg N ha^−1^ and 25–50 kg P ha^−1^. The PCA was performed using FactoMineR and factoextra packages in R software^100^. The number of PCs was selected based on the eigenvalue. The PCs that had eigenvalues ≥ 1 were kept, and 70% or greater of the total variability had to be expressed by the selected PCs. Afterward, the correlations between the selected PCs and the observed variables could be explained with factor loading. The factor loadings were estimated based on equation (1):

1 $$ {\text{Factor loadings}} = {\text{Eigenvectors}} \times \sqrt {{\text{Eigenvalue}}} $$

A factor loading of > 0.3 was considered to be significant, > 0.4 was considered more significant, and > 0.50 was considered very significant according to Lawley and Maxwell^101^. The biplot graphic showing PC1 (axis x) and PC2 (axis y) were plotted by selecting the top 100 contributing individuals.

### Research involving plants

The use of plants parts in the present study complies with international guidelines (IUCN Policy Statement on Research Involving Species at Risk of Extinction and the Convention on the Trade in Endangered Species of Wild Fauna and Flora).

## Supplementary Information

Supplementary Information.
