# Supplementary material for: Impacts of corn stover management and fertilizer application on soil nutrient availability and enzymatic activity
Source: Sci Rep. 2022 Feb 7;12:1985. doi: 10.1038/s41598-022-06042-9 (PMC8821671; doi:10.1038/s41598-022-06042-9)

**Supporting information**

**Journal: Scientific Reports**

**Impacts of corn stover management and fertilizer application on soil nutrient availability and enzymatic activity**

Fernando S. Galindo^1*^, Jeffrey S. Strock^2^, Paulo H. Pagliari^2^

^1^ University of São Paulo, Center for Nuclear Energy in Agriculture, Piracicaba, 13416-000, Brazil

^2^ Department of Soil, Water, and Climate, University of Minnesota. Southwest Research and Outreach Center. 23669 130th St. Lamberton, MN 56152.

* For correspondence: fsgalindo@usp.br (FS Galindo)

Mailing address: University of Sao Paulo, Centre for Nuclear Energy in Agriculture, Avenida Centenario, 303. CP 96, CEP 13416-000, Piracicaba, Brazil.

Phone number: +55 19 3429 4844

**Table captions for supporting files**

**Sup. Table 1.** Summary of statistical analysis for nitrate (NO_3_^-^), ammonium (NH_4_^+^), Bray-1 P, phosphatase, sulfatase, glucosidase and fluorescein diacetate hydrolysis (FDA) in 1^st^ and 2^nd^ samplings in 2013 as a function of residue management, N and P_2_O_5_ application rates. Nrate and Prate, and Nquad and Pquad refers to linear and non-linear models for N and P_2_O_5_, respectively. Glucosidase = β-glucosidase; Phosphatase = Alkaline phosphatase; Sulfatase = Arylsulfatase. Data were analyzed at *P* ≤ 0.05 using the mixed procedure of SAS 9.4^88^.

**Sup. Table 2.** Summary of statistical analysis for nitrate (NO_3_^-^), ammonium (NH_4_^+^), Bray-1 P, phosphatase, sulfatase, glucosidase and fluorescein diacetate hydrolysis (FDA) in 1^st^, 2^nd^ and 3^rd^ samplings in 2014 as a function of residue management, N and P_2_O_5_ application rates. Nrate and Prate, and Nquad and Pquad refers to linear and non-linear models for N and P_2_O_5_, respectively. Glucosidase = β-glucosidase; Phosphatase = Alkaline phosphatase; Sulfatase = Arylsulfatase.

Data were analyzed at *P* ≤ 0.05 using the mixed procedure of SAS 9.4^88^.

**Sup. Table 3.** Summary of statistical analysis for nitrate (NO_3_^-^), ammonium (NH_4_^+^), Bray-1 P, phosphatase, sulfatase, glucosidase and fluorescein diacetate hydrolysis (FDA) in 1^st^, 2^nd^ and 3^rd^ samplings in 2015 as a function of residue management, N and P_2_O_5_ application rates. Nrate and Prate, and Nquad and Pquad refers to linear and non-linear models for N and P_2_O_5_, respectively. Glucosidase = β-glucosidase; Phosphatase = Alkaline phosphatase; Sulfatase = Arylsulfatase.

Data were analyzed at *P* ≤ 0.05 using the mixed procedure of SAS 9.4^88^.

**Figure captions for supporting files**

**Sup. Figure 1.** Rainfall, maximum and minimum temperatures obtained from the weather station located in Lamberton, Minnesota during the corn cultivation.

**Sup. Table 1.**

| *P*-value | **2013** | | | | | | |
| --- | --- | --- | --- | --- | --- | --- | --- |
|  | 1^st^ sampling | | | | | | |
| **Effect** | **NO_3_^-^** | **NH_4_^+^** | **Bray-1 P** | **Phosphatase** | **Sulfatase** | **Glucosidase** | **FDA** |
| Residue management (R) | 0.705 | 0.032 | 0.756 | 0.395 | 0.725 | 0.165 | 0.896 |
| Nrate (N) | 0.927 | 0.325 | 0.167 | 0.697 | 0.539 | 0.575 | 0.891 |
| N × R | 0.655 | 0.297 | 0.175 | 0.936 | 0.793 | 0.837 | 0.742 |
| Nquad (Nq) | 0.001 | 0.662 | 0.173 | 0.773 | 0.488 | 0.837 | 0.485 |
| Nq × R | 0.598 | 0.061 | 0.198 | 0.778 | 0.451 | 0.952 | 0.432 |
| Prate (P) | 0.229 | 0.316 | 0.607 | 0.707 | 0.225 | 0.739 | 0.993 |
| P × R | 0.275 | 0.818 | 0.690 | 0.571 | 0.056 | 0.459 | 0.580 |
| Pquad (Pq) | 0.184 | 0.292 | 0.639 | 0.868 | 0.101 | 0.710 | 0.786 |
| Pq × R | 0.171 | 0.981 | 0.642 | 0.510 | 0.111 | 0.974 | 0.626 |
| N × P | 0.803 | 0.711 | 0.781 | 0.933 | 0.310 | 0.660 | 0.259 |
| N × P × R | 0.515 | 0.212 | 0.702 | 0.597 | 0.124 | 0.179 | 0.449 |
| *P*-value | 2^nd^ sampling | | | | | | |
| **Effect** | **NO_3_^-^** | **NH_4_^+^** | **Bray-1 P** | **Phosphatase** | **Sulfatase** | **Glucosidase** | **FDA** |
| Residue management (R) | 0.242 | 0.171 | 0.736 | 0.170 | 0.853 | 0.034 | 0.001 |
| Nrate (N) | 0.186 | 0.169 | 0.465 | 0.810 | 0.636 | 0.449 | 0.226 |
| N × R | 0.413 | 0.411 | 0.786 | 0.157 | 0.087 | 0.031 | 0.353 |
| Nquad (Nq) | 0.001 | 0.147 | 0.459 | 0.766 | 0.270 | 0.968 | 0.180 |
| Nq × R | 0.007 | 0.169 | 0.853 | 0.507 | 0.392 | 0.186 | 0.954 |
| Prate (P) | 0.862 | 0.200 | 0.530 | 0.777 | 0.234 | 0.440 | 0.210 |
| P × R | 0.062 | 0.502 | 0.162 | 0.060 | 0.368 | 0.753 | 0.730 |
| Pquad (Pq) | 0.884 | 0.029 | 0.137 | 0.977 | 0.220 | 0.620 | 0.228 |
| Pq × R | 0.111 | 0.272 | 0.341 | 0.061 | 0.664 | 0.971 | 0.390 |
| N × P | 0.363 | 0.871 | 0.858 | 0.949 | 0.931 | 0.676 | 0.800 |
| N × P × R | 0.004 | 0.696 | 0.021 | 0.169 | 0.055 | 0.437 | 0.435 |

**Sup. Table 2.**

| *P*-value | **2014** | | | | | | |
| --- | --- | --- | --- | --- | --- | --- | --- |
|  | 1^st^ sampling | | | | | | |
| **Effect** | **NO_3_^-^** | **NH_4_^+^** | **Bray-1 P** | **Phosphatase** | **Sulfatase** | **Glucosidase** | **FDA** |
| Residue management (R) | 0.667 | 0.926 | 0.695 | 0.999 | 0.786 | 0.314 | 0.304 |
| Nrate (N) | 0.002 | 0.001 | 0.034 | 0.553 | 0.901 | 0.490 | 0.812 |
| N × R | 0.316 | 0.251 | 0.909 | 0.113 | 0.624 | 0.618 | 0.362 |
| Nquad (Nq) | 0.001 | 0.143 | 0.043 | 0.676 | 0.984 | 0.490 | 0.712 |
| Nq × R | 0.373 | 0.215 | 0.826 | 0.268 | 0.756 | 0.841 | 0.210 |
| Prate (P) | 0.063 | 0.057 | 0.029 | 0.374 | 0.716 | 0.317 | 0.138 |
| P × R | 0.033 | 0.751 | 0.934 | 0.093 | 0.441 | 0.448 | 0.062 |
| Pquad (Pq) | 0.131 | 0.052 | 0.399 | 0.841 | 0.875 | 0.060 | 0.311 |
| Pq × R | 0.076 | 0.900 | 0.419 | 0.204 | 0.039 | 0.887 | 0.060 |
| N × P | 0.002 | 0.165 | 0.301 | 0.155 | 0.502 | 0.004 | 0.549 |
| N × P × R | 0.035 | 0.763 | 0.187 | 0.273 | 0.140 | 0.239 | 0.300 |
| *P*-value | 2^nd^ sampling | | | | | | |
| **Effect** | **NO_3_^-^** | **NH_4_^+^** | **Bray-1 P** | **Phosphatase** | **Sulfatase** | **Glucosidase** | **FDA** |
| Residue management (R) | 0.753 | 0.609 | 0.802 | 0.419 | 0.985 | 0.245 | 0.591 |
| Nrate (N) | 0.001 | 0.001 | 0.798 | 0.715 | 0.553 | 0.023 | 0.219 |
| N × R | 0.698 | 0.036 | 0.642 | 0.814 | 0.358 | 0.255 | 0.757 |
| Nquad (Nq) | 0.008 | 0.454 | 0.841 | 0.679 | 0.656 | 0.140 | 0.262 |
| Nq × R | 0.814 | 0.198 | 0.899 | 0.888 | 0.528 | 0.432 | 0.272 |
| Prate (P) | 0.001 | 0.712 | 0.448 | 0.929 | 0.607 | 0.461 | 0.293 |
| P × R | 0.807 | 0.056 | 0.985 | 0.618 | 0.550 | 0.085 | 0.085 |
| Pquad (Pq) | 0.001 | 0.414 | 0.114 | 0.880 | 0.347 | 0.269 | 0.419 |
| Pq × R | 0.977 | 0.060 | 0.563 | 0.450 | 0.146 | 0.050 | 0.094 |
| N × P | 0.001 | 0.740 | 0.992 | 0.570 | 0.455 | 0.583 | 0.573 |
| N × P × R | 0.410 | 0.094 | 0.602 | 0.709 | 0.107 | 0.958 | 0.507 |
| *P*-value | 3^rd^ sampling | | | | | | |
| **Effect** | **NO_3_^-^** | **NH_4_^+^** | **Bray-1 P** | **Phosphatase** | **Sulfatase** | **Glucosidase** | **FDA** |
| Residue management (R) | 0.409 | 0.610 | 0.656 | 0.034 | 0.602 | 0.070 | 0.023 |
| Nrate (N) | 0.001 | 0.025 | 0.075 | 0.171 | 0.061 | 0.701 | 0.809 |
| N × R | 0.409 | 0.479 | 0.778 | 0.236 | 0.465 | 0.498 | 0.861 |
| Nquad (Nq) | 0.001 | 0.770 | 0.047 | 0.168 | 0.062 | 0.810 | 0.782 |
| Nq × R | 0.364 | 0.763 | 0.741 | 0.363 | 0.301 | 0.388 | 0.885 |
| Prate (P) | 0.001 | 0.964 | 0.249 | 0.872 | 0.267 | 0.866 | 0.459 |
| P × R | 0.428 | 0.630 | 0.272 | 0.410 | 0.050 | 0.907 | 0.225 |
| Pquad (Pq) | 0.001 | 0.785 | 0.228 | 0.735 | 0.296 | 0.456 | 0.210 |
| Pq × R | 0.349 | 0.558 | 0.149 | 0.621 | 0.276 | 0.940 | 0.384 |
| N × P | 0.001 | 0.554 | 0.506 | 0.568 | 0.648 | 0.204 | 0.271 |
| N × P × R | 0.654 | 0.009 | 0.919 | 0.396 | 0.604 | 0.872 | 0.797 |

**Sup. Table 3.**

| *P*-value | **2015** | | | | | | |
| --- | --- | --- | --- | --- | --- | --- | --- |
|  | 1^st^ sampling | | | | | | |
| **Effect** | **NO_3_^-^** | **NH_4_^+^** | **Bray-1 P** | **Phosphatase** | **Sulfatase** | **Glucosidase** | **FDA** |
| Residue management (R) | 0.666 | 0.096 | 0.893 | 0.760 | 0.595 | 0.924 | 0.164 |
| Nrate (N) | 0.423 | 0.006 | 0.167 | 0.307 | 0.395 | 0.229 | 0.200 |
| N × R | 0.761 | 0.021 | 0.430 | 0.014 | 0.662 | 0.538 | 0.458 |
| Nquad (Nq) | 0.021 | 0.121 | 0.125 | 0.577 | 0.638 | 0.309 | 0.298 |
| Nq × R | 0.928 | 0.027 | 0.638 | 0.034 | 0.562 | 0.894 | 0.299 |
| Prate (P) | 0.832 | 0.175 | 0.001 | 0.052 | 0.573 | 0.358 | 0.880 |
| P × R | 0.430 | 0.548 | 0.428 | 0.098 | 0.565 | 0.649 | 0.086 |
| Pquad (Pq) | 0.725 | 0.136 | 0.003 | 0.545 | 0.697 | 0.042 | 0.845 |
| Pq × R | 0.412 | 0.422 | 0.107 | 0.155 | 0.061 | 0.908 | 0.124 |
| N × P | 0.020 | 0.892 | 0.714 | 0.010 | 0.717 | 0.001 | 0.183 |
| N × P × R | 0.390 | 0.953 | 0.592 | 0.359 | 0.121 | 0.236 | 0.377 |
| *P*-value | 2^nd^ sampling | | | | | | |
| **Effect** | **NO_3_^-^** | **NH_4_^+^** | **Bray-1 P** | **Phosphatase** | **Sulfatase** | **Glucosidase** | **FDA** |
| Residue management (R) | 0.109 | 0.150 | 0.855 | 0.973 | 0.884 | 0.001 | 0.202 |
| Nrate (N) | 0.513 | 0.026 | 0.382 | 0.877 | 0.094 | 0.016 | 0.317 |
| N × R | 0.565 | 0.106 | 0.504 | 0.979 | 0.254 | 0.770 | 0.717 |
| Nquad (Nq) | 0.392 | 0.784 | 0.484 | 0.857 | 0.759 | 0.159 | 0.382 |
| Nq × R | 0.709 | 0.306 | 0.867 | 0.906 | 0.501 | 0.913 | 0.282 |
| Prate (P) | 0.731 | 0.014 | 0.004 | 0.279 | 0.172 | 0.636 | 0.733 |
| P × R | 0.143 | 0.076 | 0.858 | 0.447 | 0.949 | 0.738 | 0.152 |
| Pquad (Pq) | 0.920 | 0.054 | 0.682 | 0.271 | 0.098 | 0.371 | 0.999 |
| Pq × R | 0.330 | 0.074 | 0.559 | 0.218 | 0.239 | 0.499 | 0.276 |
| N × P | 0.011 | 0.457 | 0.454 | 0.774 | 0.391 | 0.409 | 0.852 |
| N × P × R | 0.102 | 0.127 | 0.959 | 0.609 | 0.016 | 0.732 | 0.303 |
| *P*-value | 3^rd^ sampling | | | | | | |
| **Effect** | **NO_3_^-^** | **NH_4_^+^** | **Bray-1 P** | **Phosphatase** | **Sulfatase** | **Glucosidase** | **FDA** |
| Residue management (R) | 0.479 | 0.550 | 0.658 | 0.236 | 0.833 | 0.208 | 0.002 |
| Nrate (N) | 0.261 | 0.036 | 0.357 | 0.059 | 0.200 | 0.387 | 0.265 |
| N × R | 0.544 | 0.973 | 0.863 | 0.342 | 0.135 | 0.330 | 0.235 |
| Nquad (Nq) | 0.171 | 0.983 | 0.125 | 0.063 | 0.165 | 0.802 | 0.450 |
| Nq × R | 0.527 | 0.525 | 0.914 | 0.627 | 0.125 | 0.380 | 0.356 |
| Prate (P) | 0.016 | 0.858 | 0.001 | 0.514 | 0.769 | 0.418 | 0.006 |
| P × R | 0.535 | 0.441 | 0.132 | 0.612 | 0.031 | 0.843 | 0.661 |
| Pquad (Pq) | 0.072 | 0.768 | 0.631 | 0.146 | 0.777 | 0.195 | 0.004 |
| Pq × R | 0.333 | 0.554 | 0.034 | 0.888 | 0.289 | 0.931 | 0.535 |
| N × P | 0.020 | 0.433 | 0.149 | 0.328 | 0.847 | 0.598 | 0.954 |
| N × P × R | 0.664 | 0.011 | 0.448 | 0.278 | 0.749 | 0.797 | 0.442 |

**Sup. Figure 1.**


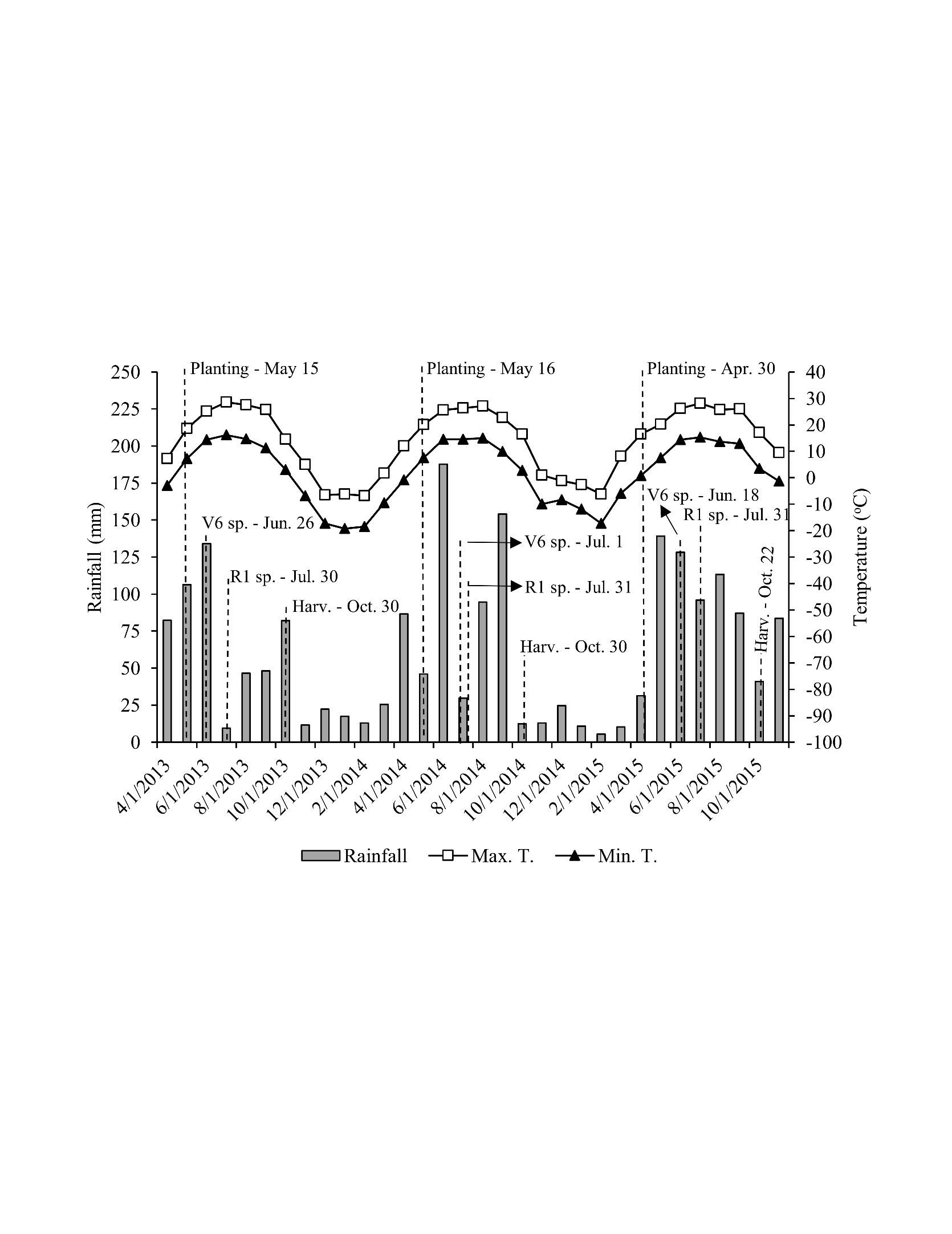

Supplement: Supplementary file 1 — Supplementary Information. [file 41598_2022_6042_MOESM1_ESM.docx]
